# A novel machine learning-based programmed cell death-related clinical diagnostic and prognostic model associated with immune infiltration in endometrial cancer

**DOI:** 10.3389/fonc.2023.1224071

**Published:** 2023-07-18

**Authors:** Jian Xiong, Junyuan Chen, Zhongming Guo, Chaoyue Zhang, Li Yuan, Kefei Gao

**Affiliations:** ^1^ Department of Obstetrics and Gynaecology, Guangzhou Women and Children’s Medical Center, Guangzhou Medical University, Guangzhou, China; ^2^ China Medical University, Shenyang, China; ^3^ Department of Pathology, Guangzhou Women and Children’s Medical Center, Guangzhou Medical University, Guangzhou, China

**Keywords:** LASSO, cell death, endometrial cancer, signature, cell assay, immune infiltration

## Abstract

**Background:**

To explore the underlying mechanism of programmed cell death (PCD)-related genes in patients with endometrial cancer (EC) and establish a prognostic model.

**Methods:**

The RNA sequencing data (RNAseq), single nucleotide variation (SNV) data, and corresponding clinical data were downloaded from TCGA. The prognostic PCD-related genes were screened and subjected to consensus clustering analysis. The two clusters were compared by weighted correlation network analysis (WGCNA), immune infiltration analysis, and other analyses. The least absolute shrinkage and selection operator (LASSO) algorithm was used to construct the PCD-related prognostic model. The biological significance of the PCD-related gene signature was evaluated through various bioinformatics methods.

**Results:**

We identified 43 PCD-related genes that were significantly related to prognoses of EC patients, and classified them into two clusters *via* consistent clustering analysis. Patients in cluster B had higher tumor purity, higher T stage, and worse prognoses compared to those in cluster A. The latter generally showed higher immune infiltration. A prognostic model was constructed using 11 genes (GZMA, ASNS, GLS, PRKAA2, VLDLR, PRDX6, PSAT1, CDKN2A, SIRT3, TNFRSF1A, LRPPRC), and exhibited good diagnostic performance. Patients with high-risk scores were older, and had higher stage and grade tumors, along with worse prognoses. The frequency of mutations in PCD-related genes was correlated with the risk score. LRPPRC, an adverse prognostic gene in EC, was strongly correlated with proliferation-related genes and multiple PCD-related genes. LRPPRC expression was higher in patients with higher clinical staging and in the deceased patients. In addition, a positive correlation was observed between LRPPRC and infiltration of multiple immune cell types.

**Conclusion:**

We identified a PCD-related gene signature that can predict the prognosis of EC patients and offer potential targets for therapeutic interventions.

## 1 Introduction

Endometrial cancer (EC) was the sixth most common cancer diagnosed in women in 2020, with a total of 417,000 new cases documented worldwide. The median age of diagnosis of EC patients is 61 years, and the lifetime risk of EC is around 3% (1). EC is associated with a high mortality rate, and over 76,000 women die annually as a result of EC (2). The mortality rate due to EC further increases with advanced tumor stage, invasive histology, and metastasis (3). There is currently a paucity of biomarkers or models that can effectively predict the prognosis and survival of EC patients (4). Therefore, it is crucial to elucidate the molecular mechanisms underlying EC occurrence and progression in order to identify novel prognostic markers and therapeutic targets.

Cell death plays a crucial role in several biological processes, and can be classified as programmed cell death (PCD) and accidental cell death (ACD) (5). PCD is the culmination of ordered, gene-controlled pathways following the spontaneous loss of cellular function, whereas ACD is an uncontrolled process that is triggered in response to certain harmful stimuli. PCD is primarily responsible for maintaining intracellular homeostasis (6). Various types of PCD have been documented so far, including autophagy-dependent cell death, necroptosis, apoptosis, ferroptosis, pyroptosis, entosis, parthanatos, NETosis, alkaliptosis, lysosome-dependent cell death (LCD), and oxeiptosis (7).

Lysosomal degradation during autophagy-mediated cell death facilitates metabolic adaptation and nutrient recycling (8). Necrosis has long been considered an involuntary form of cell death, although recent evidence indicates that necrosis can be initiated and sustained, resulting in necroptosis that centers around the formation of necrosomes (9, 10). Apoptosis is an intrinsic mechanism for eliminating damaged cells and involves a series of events including condensation, nucleolysis, and nuclear fragmentation, which culminate in the engulfment of apoptotic vesicles by macrophages (11). Ferroptosis is characterized by the accumulation of lipid hydroperoxides in an iron-dependent manner that ultimately reaches a lethal threshold (12, 13). Cuproptosis is a recently identified mode of PCD that is triggered by copper imbalance and is closely associated with disease progression (14). Pyroptosis is an inflammatory form of PCD that is typified by the creation of membrane pores that compromise cellular integrity, eventually leading to cell rupture (15). Entosis is a form of cell “cannibalism” wherein one live cell is engulfed and lysed by another cell without the activation of the apoptotic pathway (16). Parthanatos is induced by excessive activation of the nuclease PARP-1 (17), an RNA polymerase (RP) that interacts with and activates DNA or RNA, leading to replication or repair of damaged DNA and proteins. NETosis is initiated by the release of neutrophil extracellular traps (NETs), i.e., interconnected structures that cells release in response to infection or injury (18). Alkaliptosis, a newly recognized type of PCD, heavily relies on the intracellular alkalization process (19). LCD is dependent on hydrolase, which facilitates lysosomal transport to the cytoplasm by means of membrane penetration. This process is regulated by intracellular signaling systems and membrane proteins (20). KEAP1, a detector of reactive oxygen species (ROS), has recently been shown to be involved in a unique type of cell death known as oxygen apoptosis (21).

Mutations in the PCD pathways have been detected in the early stages of cancer, which endow the tumor cells with resistance to anti-cancer treatments (7). Therefore, targeted interventions that activate PCD pathways using single or combined therapies are an effective anti-cancer strategy. For instance, the FDA-approved BCL-2 inhibitor triggers apoptosis in lymphoma cells (22). In addition, activation of GSDME-mediated pyroptosis has proved to be highly effective against many cancers (23). Cancer cells can resist PD-1/PD-L1 checkpoint inhibition by blocking ferroptosis through the regulation of ferritin and other proteins (24), indicating that activation of the ferroptosis pathway in these cells can sensitize them to PD-1/PD-L1 blockers.

In this study, we used bioinformatic approaches to establish a PCD-related prognostic gene signature for EC and found that higher risk scores were associated with a worse prognosis. Our findings provide new insights into the molecular basis of EC progression. Furthermore, the genes associated with PCD have significant potential as prognostic biomarkers and therapeutic targets for EC.

## 2 Materials and methods

### 2.1 Data acquisition and preprocessing

The RNA sequencing (RNAseq), single nucleotide variation (SNV), and relevant clinical data of 544 tumor samples and 35 normal samples from TCGA-UCEC (Uterine Corpus Endometrial Carcinoma) were downloaded through the UCSC XENA website (https://xenabrowser.net/datapages/) and Sangerbox website (http://www.sangerbox.com/). TCGA-CESC (168 tumor samples) and TCGA-BRCA (1057 tumor samples) were downloaded from the UCSC XENA website (https://xenabrowser.net/datapages/) for external validation. The RNAseq data was transformed into fragments per kilobase of exon model per million mapped fragments (FPKM) format. In addition, the curated transcriptome data, SNV data, and corresponding clinical data of 12,591 patients across 32 cancer types were also retrieved from the UCSC XENA website (http://xena.ucsc.edu/). All transcriptomic data were transformed to the FPKM format for downstream analysis.

### 2.2 Screening of prognostic PCD-related genes, consensus clustering, and immune infiltration

A list of PCD-related genes (**Supplementary Table S1**) was obtained through literature review and manual searching (7), and these genes were extracted from the TCGA-UCEC cohort. The prognostically relevant genes were screened through univariate Cox regression analysis using the “survival” package, and subjected to consensus clustering using the “ConsensusClusterPlus” package. The patients were accordingly divided into two clusters. The “limma” and “estimate” packages were used for scoring immune filtration, and the results were visualized using the “ComplexHeatmap”, “gplots”, “ggplot2”, “RColorBrewer” and “oompaBase” packages. The immune cell populations were characterized on the basis of classical markers, including immunoglobulin G (IgG), hematopoietic cell kinase (HCK), major histocompatibility complex class II (MHC-II), lymphocyte-specific kinase (LCK), activation transcription 1 (STAT1), interferons, TNF and B7-CD28 (CD28) (25). The scores of the immune cell subsets in both clusters were evaluated by ssGSEA. Weighted correlation network analysis (WGCNA) was performed using the “WGCNA” and “limma” packages, with cluster, age, stage, grade, and tumor mutation burden (TMB) as the factors. The gene modules with the strongest correlations with each factor were screened using abline=60 and soft threshold=4 as the criteria. Finally, the PCD-related genes were functionally annotated by GO analysis.

### 2.3 Construction and validation of PCD-related genes signature

The “glmnet” package was used for LASSO analysis to select the PCD-related genes for the prognostic model. Although overfit potential generally exists in machine learning-based models, feature selection can effectively solve the overfitting problem because it can select the most relevant features from the original feature set, thus reducing the complexity and noise interference of the model. Common feature selection methods include Filter method, Wrapper method, and Embedded method. Among them, the Filter method sorts and filters according to the correlation between features and target variables. The wrapper method, by constantly trying different feature subsets, uses the model itself to evaluate and select the optimal subset. Embedded methods use feature selection as part of the model training process, such as LASSO and ridge regression. As our model is exactly based on the LASSO algorithm, the overfit potential could be avoided to some extent. The risk score was calculated by multiplying the expression of each gene with its respective coefficient:


(1) $$\text{Riskscore }={\sum_{i=1}^{n}[\exp ression\;value\;of\;gene}_{i}*\beta_{i}]$$

The variable “n” represents the number of genes included in the signature, and the variable “β” denotes the coefficient assigned to each gene obtained from LASSO regression.

Risk score= [expression value of GZMA × (-0.116286998)] + [expression value of ASNS × (0.127252486)] + [expression value of GLS × (0.042654301)] + [expression value of PRKAA2 × (0.1310519)] + [expression value of VLDLR × (-0.114179696)] + [expression value of PRDX6 × (0.016522634)] + [expression value of PSAT1 × (0.016313951)] + [expression value of CDKN2A × (0.116960742)] + [expression value of SIRT3 × (-0.180170396)] + [expression value of TNFRSF1A × (-0.026584716)] +[expression value of LRPPRC × (0.055918364)]

According to the median risk score, patients were divided into high-risk and low-risk groups. The “survival” package was used to perform proportional hazards assumption testing and survival regression, and the “survminer” and “ggplot2” packages were used to plot the graphs. The diagnostic capability of PCD-related gene signature in the TCGA-UCEC cohort was evaluated by receiver operational characteristic (ROC) analysis using the “timeROC” package, and the results were visualized with the “ggplot2” package. All the samples with survival information were included in the ROC analysis. The number of samples was 544. The area under the curve (AUC) was calculated, and 0.7 was the cut-off for satisfactory diagnostic performance. The “ggplot2” package was used to visualize the risk factor plot. The prognostic model was tested on 32 types of cancer (**Supplementary Table S2**) using the “ggplot2”, “data.table”, “survival”, “cowplot” and “ggpub” packages. The prognostic endpoints of the patients included disease-specific survival (DSS), overall survival (OS), and progression-free interval (PFI).

### 2.4 Analysis of single-nucleotide variations of PCD-related genes

Pan-cancer analysis on the top 10 mutated genes in the signature using the “ggplot2”, “data.table”, “cowplot”, “ggpubr”, “GSVA”, “SimDesign” and “tidyr” packages. The mutation frequencies of the PCD-related genes in the different organs were also calculated.

### 2.5 Comparison of risk groups

The “ComplexHeatmap” package was used to create a heatmap showing the relationship between the risk score and the clinical factors like age, grade, and stage. Gene set enrichment analysis (GSEA) was performed on the high-risk and low-risk groups, and the top 5 pathways in each group were identified. The “RColorBrewer” package was used to visualize the results. The “oncoPredict” package was used for drug sensitivity analysis.

### 2.6 Construction and validation of a prognostic nomogram

The “survival” package was used to perform proportional hazards assumption testing and Cox regression analysis, and a nomogram consisting of age, stage, grade, and the PCD risk score was constructed using the “rms” package. The 1-, 3- and 5-year survival were predicted using the nomogram. Calibration curves for 1-, 3- and 5-year survival were plotted using the “rms” package. All the samples with survival information were included in the ROC analysis. The number of samples was 544.

### 2.7 Correlation between PCD-related genes, proliferation genes, and immune cell phenotypes

The correlation between the PCD-related genes and proliferation-related genes (*WNT5A, PCNA, MKI67, CTNNB1*, and *CDH1*) was analyzed using the “ggplot2” package. *LRPPRC* was identified as the gene of interest. The “survival” and “survminer” packages were used to evaluate the prognostic relevance of *LRPPRC*, and analyze its expression across different clinical stages and OS events. The “igraph” and “ggraph” packages were used to analyze the pairwise correlation of genes within the PCD-related gene signature. Finally, the “ggplot2” package was used to visualize the association between LRPPRC and the infiltration of 24 immune cell types infiltration through Spearman correlation analysis.

### 2.8 Statistical analysis

R software (4.1.3) was used for statistical analyses. T-test was used to compare the data between the two groups. P value< 0.05 was considered statistically significant.

## 3 Results

### 3.1 Identification and clustering of prognostic PCD-related genes

**Figure 1:**
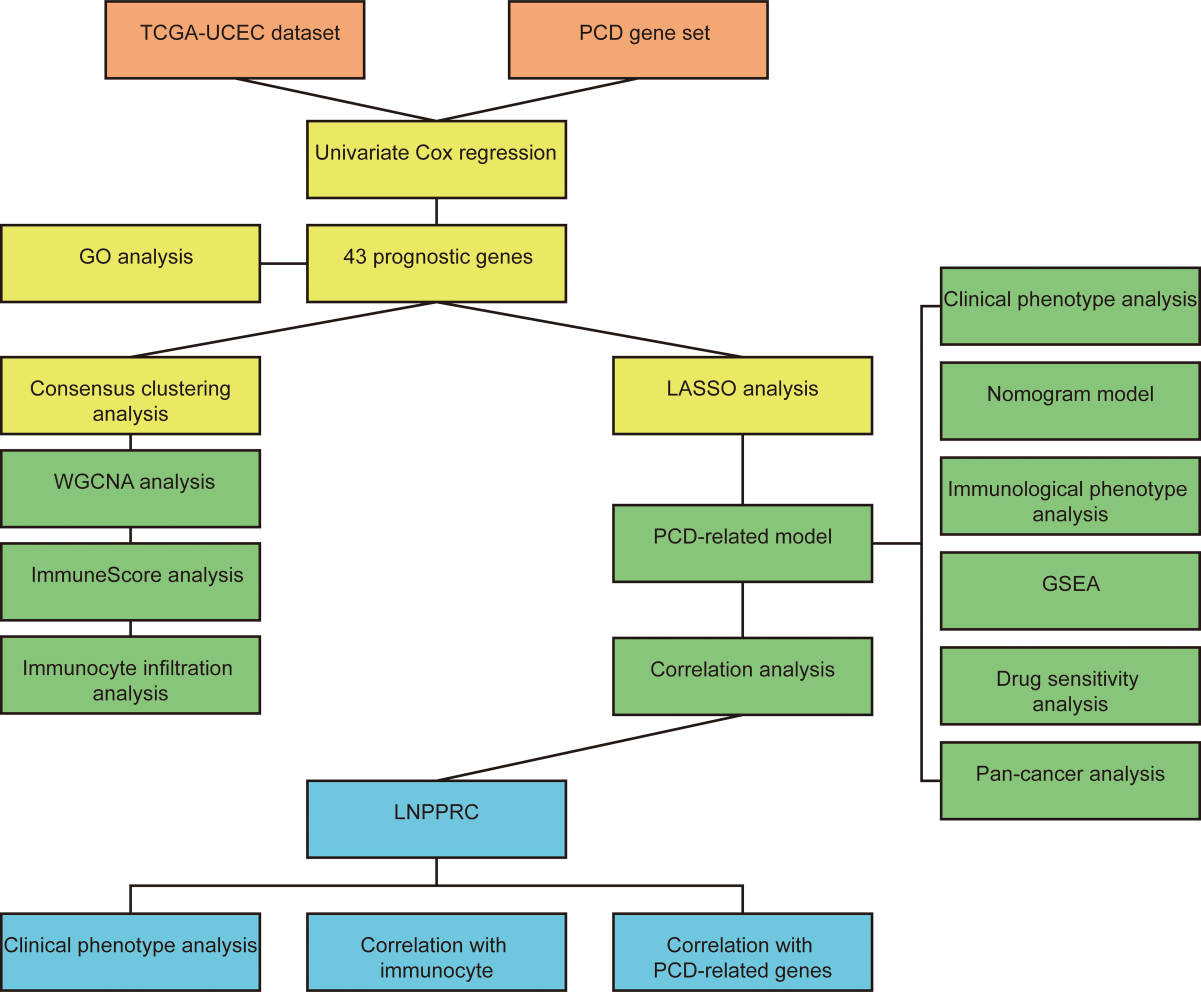
The workflow chart of the study.

**Figure 2:**
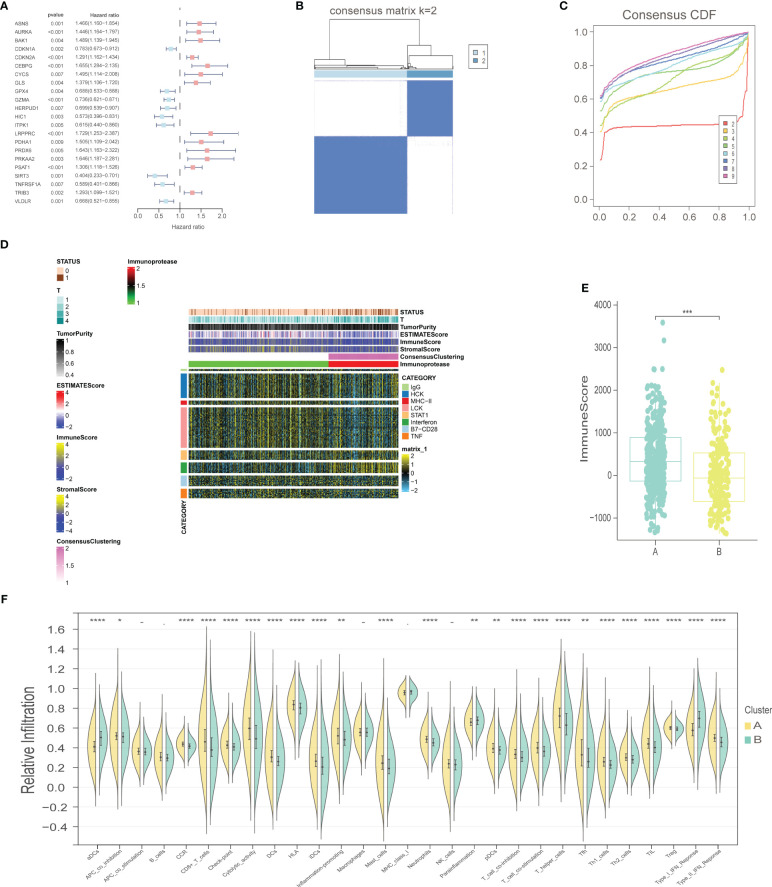
Selection of prognostic-related PCD-related genes, consensus clustering analysis, and immune-related analysis. **(A)** Forest plot of PCD-related genes with prognostic significance at p<0.01. **(B)** Consensus matrix, k=2. **(C)** Consensus CDF. **(D)** Heat map of immune scores. **(E)** ImmuneScore boxplot of two clusters. **(F)** Violin plot of immune infiltration analysis for two clusters. "*" represents p < 0.05; "**" represents p < 0.01; "***" represents p < 0.001; "****" represents p < 0.0001.

**Figure 3:**
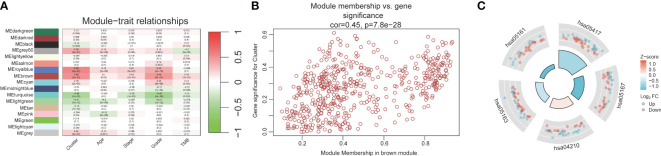
WGCNA and GO analysis. **(A)** Heat map of module-trait relationships. **(B)** Scatter plot of module membership versus gene significance. **(C)** GO analysis for 43 prognostic PCD-related genes.

The workflow of our study is illustrated in **Figure 1**. We identified 43 PCD-related genes that were significantly correlated to the prognosis of EC patients (**Supplementary Table S3**), and the Forest plot of the genes with p<0.01 is shown in **Figure 2A**. Consensus clustering analysis of these 43 genes with 2-9 clusters showed that dividing the samples into two clusters resulted in better distinction (**Figures 2B, C**). Patients in Cluster B had higher tumor purity, more advanced T stage, and worse prognosis compared to those in Cluster A. The latter generally had higher ESTIMATEScore, ImmuneScore, and StromalScore (**Supplementary Table S4**). Furthermore, Cluster A was associated with high expression levels of *IgG, HCK, MHC-II, LCK, B7-CD28*, and *TNF*, whereas *STAT1* and *IFN* were overexpressed in Cluster B (**Figures 2D, E**). Subsequently, ssGSEA showed distinct immune cell infiltration patterns of Cluster A and Cluster B (p<0.05). For instance, CD8^+^ T cells, cytolytic activity, inflammation-promoting, T-cell co-stimulation, etc. were significantly higher in Cluster A compared with Cluster B, whereas the latter had higher levels of aDCs, para inflammation, and Type I IFN response (**Figure 2F**). The results of WGCNA indicated that the brown module had a high degree of positive correlation with both Cluster and Grade, with correlation coefficients of 0.49 (p<0.05). You could see that the brown module had a high degree of positive correlation (0.49) with both Cluster and Grade. In the meanwhile, other modules were almost poorly or negatively correlated with Cluster and Grade. That is to say, the brown module shows high specificity for Cluster and Grade. Although the royal blue module had the strongest correlation with Cluster (with a related coefficient of 0.56, p<0.05), the low number of genes in the module was not representative. Therefore, we selected the brown module for further analysis (**Figure 3A**). The correlation scatter plot of Module Membership in the brown module (MM) and Gene significance for Cluster (GS) showed a highly positive correlation (with a correlation coefficient of 0.45, p=7.8*10^-28^), which suggested that the brown module genes were upregulated in Cluster B (**Figure 3B**). GO analysis indicated that the 43 prognosis-related PCD-related genes were significantly downregulated in the “lipid and atherosclerosis” pathway (**Figure 3C**; **Table 1**).

**Table 1:** Description of ID in GO analysis.

ID	Description
hsa05417	Lipid and atherosclerosis
hsa05167	Kaposi sarcoma-associated herpesvirus infection
hsa04210	Apoptosis
hsa05163	Human cytomegalovirus infection
hsa05161	Hepatitis B

### 3.2 Construction and validation of PCD-related genes signature

**Figure 4:**
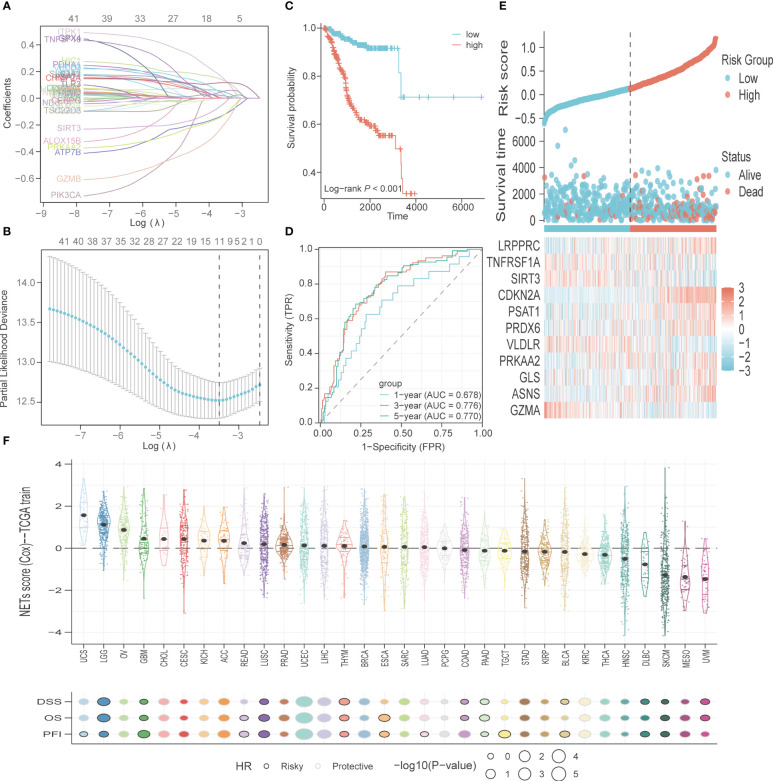
Construction and validation of PCD-related genes signature. **(A)** The variable coefficient values, logarithmic lambda values, and L1 regularization values obtained through LASSO analysis were visualized. **(B)** Lambda values, maximum likelihood numbers, and C-Index obtained from LASSO analysis were visualized. **(C)** K-M curves for OS in high-risk and low-risk patient groups. **(D)** ROC analysis to evaluate the performance of signature in 1-year, 3-year, and 5-year prognoses. **(E)** Visualization of risk factors. **(F)** The PCD risk score in other types of cancer, with patients’ prognostic endpoints including DSS, OS, and PFI.

A total of 200 PCD-related genes were incorporated in the LASSO algorithm and 11 genes (*GZMA, ASNS, GLS, PRKAA2, VLDLR, PRDX6, PSAT1, CDKN2A, SIRT3, TNFRSF1A, LRPPRC*) were finally selected to construct the prognostic gene signature (**Figures 4A, B**). The detailed information and coefficients of these genes are shown in **Supplementary Table S5**. The risk scores of the individual patients in the TCGA-UCEC cohort were calculated (see methods), and the patients were divided into high-risk and low-risk groups based on the median PCD risk score. As shown in **Figure 4C**, higher risk scores correlated with shorter OS and higher mortality rates (p<0.001). In addition, the PCD signature showed strong predictive performance for 1-, 3- and 5-year OS, with respective AUC values of 0.678, 0.776, and 0.77 (**Figure 4D**). *LRPPRC, CDKN2A, PSAT1, PRDX6, PRKAA2, GLS*, and *ASNS* were overexpressed in the high-risk group, whereas *TNFRSF1A, SIRT3, VLDLR*, and *GZMA* were highly expressed in the low-risk group (**Figure 4E**). We also tested the risk score across 32 types of cancer and found that uterine carcinosarcoma, brain lower-grade glioma, and ovarian serous cystadenocarcinoma had higher risk scores, while uveal melanoma, mesothelioma, and skin cutaneous melanoma typically had lower risk scores. The PCD risk score distribution for other cancers is shown in **Figure 4F**.

The PCD-related genes signature was further validated in TCGA-CESC and TCGA-BRCA cohorts. The risk scores of the individual patients in the TCGA-BRCA cohort were calculated, and the patients were divided into high-risk and low-risk groups based on the median PCD risk score. As shown in **Figure S1A**, higher risk scores correlated with shorter OS and higher mortality rates (p<0.001). In addition, the PCD signature showed strong predictive performance for 1-, 3- and 5-year OS, with respective AUC values of 0.661, 0.647, and 0.632 (**Figure S1B**). The calibration curves for 1-, 3- and 5-year OS showed good consistency between observed survival and predicted survival (**Figure S1C**). The risk scores of the individual patients in the TCGA-CESC cohort were also calculated, and the patients were divided into high-risk and low-risk groups based on the median PCD risk score. As shown in **Figure S1D**, higher risk scores correlated with shorter OS and higher mortality rates (p<0.01). In addition, the PCD signature showed strong predictive performance for 1-, 3- and 5-year OS, with respective AUC values of 0.765, 0.816, and 0.849 (**Figure S1E**). The calibration curves for 1-, 3- and 5-year OS showed good consistency between observed survival and predicted survival (**Figure S1F**).

### 3.3 Pan-cancer variations in PCD-related genes

**Figure 5:**
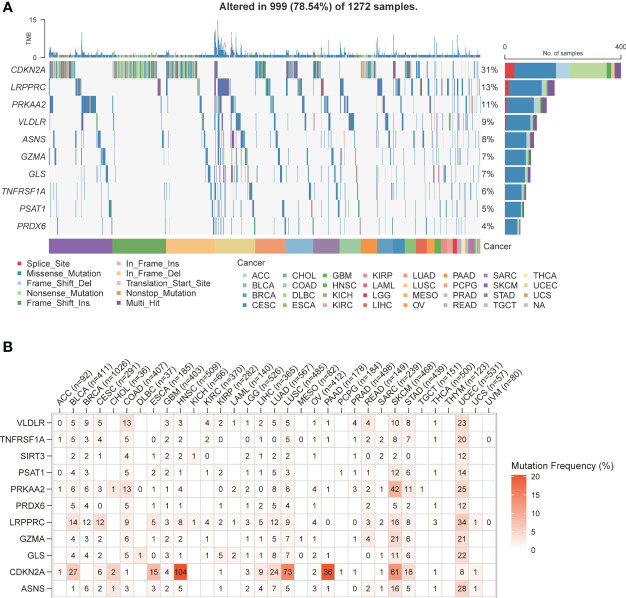
PCD-related genes SNV patterns. **(A)** Waterfall plot of PCD-related genes SNV pattern in various types of cancer. **(B)** Mutation frequency of PCD-related genes in various types of cancer.

The pan-cancer SNV profiles of the top 10 mutated genes of the PCD risk model were also analyzed. *CDKN2A* displayed higher mutation rates across multiple cancer types (31%), and other frequently mutated genes included *LRPPRC* (13%), *PRKAA2* (11%), *VLDLR* (9%), *ASNS* (8%), *GZMA* (7%), *GLS* (7%), *TNFRSF1A* (6%), *PSAT1* (5%), and *PRDX6* (4%). A missense mutation was the most frequently observed mutation type. However, the mutation types in *CDKN2A* were predominantly nonsense mutation, splice site, frameshift deletion, and multi-hit (**Figure 5A**). Furthermore, *CDKN2A* displayed higher mutation frequency in head and neck squamous cell carcinoma (104%), lung squamous cell carcinoma (73%), skin cutaneous melanoma (61%), pancreatic adenocarcinoma (36%), bladder urothelial carcinoma (27%), and lung adenocarcinoma (24%). The SNV frequency of *PRKAA2* in skin cutaneous melanoma was 42%. Also, several PCD-related genes exhibited high mutation frequency in uterine corpus endometrial carcinoma and skin cutaneous melanoma (**Figure 5B**).

### 3.4 Comprehensive analysis of two risk groups

**Figure 6:**
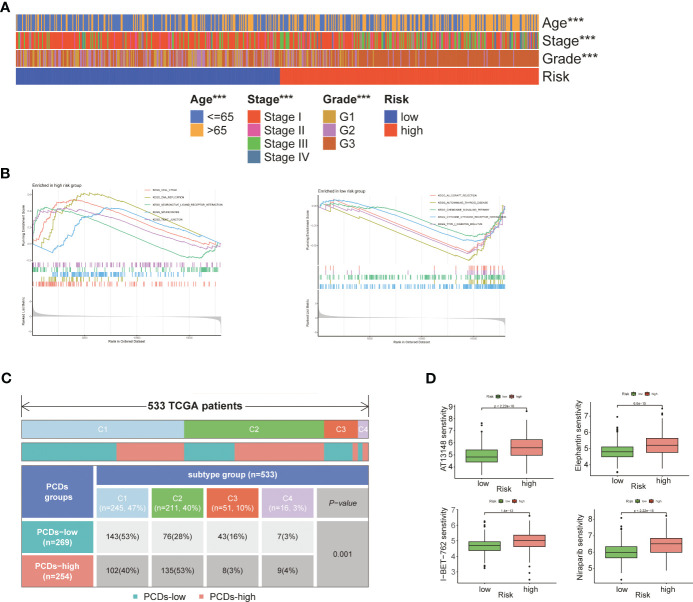
Comprehensive analysis of two risk groups. **(A)** Heat map of the correlation between risk groups and clinical characteristics. **(B)** GSEA analysis of two risk groups. **(C)** Immunotyping analysis. **(D)** Boxplot of the difference in drug sensitivity between high-risk and low-risk groups. "***" represents p < 0.001.

As shown in **Figure 6A**, patients in the high-risk group were older (p<0.001) and had more advanced tumor grade and stage (p<0.001), which was indicative of poor prognosis. GSEA on the two risk groups revealed significant enrichment of “KEGG_CELL_CYCLE” and “KEGG_DNA_REPLICATION” in the high-risk group, and that of “KEGG_CHEMOKINE_SIGNALING_PATHWAY” and “KEGG_CYTOKINE_CYTOKINE_RECEPTOR_INTERACTION” in the low-risk group (**Figure 6B**; **Supplementary Table S6**). Four subgroups were identified through immune typing analysis. The proportion of high-risk patients was higher in the c2 subgroup, while the opposite trend was observed in the other subgroups. The sample distribution of the high-risk and low-risk groups differed significantly (p<0.001) across the four subgroups (**Figure 6C**). Drug sensitivity analysis also showed significant differences between the high-risk and low-risk groups (**Supplementary Table S7**). As shown in **Figure 6D**, patients in the high-risk group showed greater sensitivity to AT3148, Elephantin, I-BET-762, and Niraparib (p<0.001).

### 3.5 Construction and validation of a prognostic nomogram

**Figure 7:**
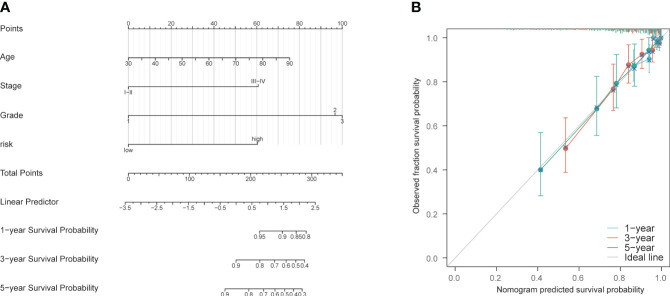
Construction and validation of nomogram model. **(A)** Nomogram prognostic model. **(B)** 1-year, 3-year, and 5-year calibration curves.

A nomogram was constructed using age, staging, grading, and the PCD risk score to predict patient survival at 1, 3, and 5 years (**Supplementary Table S8**). The scores for each risk factor were calculated, and the total score was plotted (**Figure 7A**). On this basis, this study intends to establish a correction curve to verify the applicability of the model at the PCD gene level. Our prognostic indicators predicted favorable 1,3 and 5-year survival rates (**Figure 7B**).

### 3.6 Correlation between PCD-related genes, proliferation genes, and immune cell phenotypes

**Figure 8:**
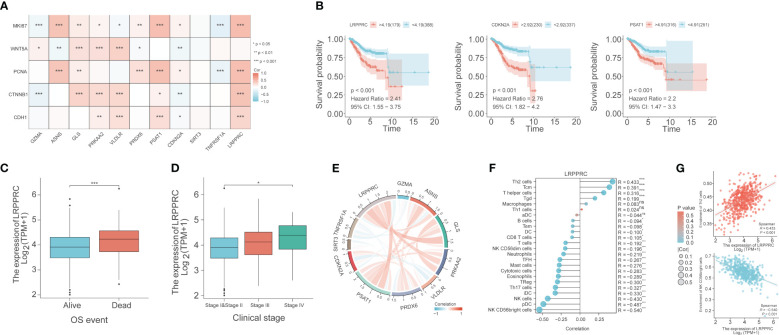
Bioinformatics analysis reveals the correlation between PCD-related genes, proliferation genes, and immune cell phenotypes. **(A)** Correlation heat map depicting the relationship between genes in the PCD-related gene signature and genes associated with cell proliferation. **(B)** Survival curves for LRPPRC, CDKN2A, and PSAT1. **(C)** Box plot of the relationship between LRPPRC and OS event. **(D)** Box plot of the relationship between LRPPRC and clinical stage. **(E)** Co-expression network within PCD-related genes. **(F)** The lollipop graph depicts the correlation between LRPPRC and immune phenotype. **(G)** Scatter plot of the correlation between LRPPRC expression and Th2 cells and CD56bright cells, respectively. "*" represents p < 0.05; "**" represents p < 0.01; "***" represents p < 0.001.

The correlation between the expression of the PCD-related genes and five proliferation-related genes, including *WNT5A, PCNA, MKI67, CTNNB1*, and *CDH1*, was also evaluated. *LRPPRC* showed a significant positive correlation with *PCNA, MKI67, CTNNB1*, and *CDH1* (p< 0.001), which indicated that LRPPRC may promote the proliferation of EC cells. *CDKN2A* was correlated to all five proliferation-related genes, with a positive correlation with MKI67 and PCNA (p<0.05), and a negative correlation with *WNT5A, CTNNB1*, and *CDH1* (p<0.01). PSAT1 was positively correlated with *PCNA, MKI67, CTNNB1* and *CDH1* (p<0.05; **Figure 8A**). High expression levels of *LRPPRC*, *CDKN2A*, and *PSAT1* were associated with poor patient prognosis (**Figure 8B**). Furthermore, *LRPPRC* was expressed at higher levels in the deceased patients compared to the surviving patients (p< 0.01; **Figure 8C**). Furthermore, *LRPPRC* was also significantly upregulated in stage IV tumors compared to stage I/II tumors (p< 0.05; **Figure 8D**). *LRPPRC* also showed a significant positive correlation with some genes of the prognostic signature, including *GLS*, *PRKAA2*, and *PSAT1*, and this correlation was stronger compared to that among the other genes (**Figure 8E**). Finally, the relationship between *LRPPRC* and the infiltration of 24 immune cells was analyzed. *LRPPRC* showed a highly positive correlation with Th2 cells (R=0.433), Tcm cells (R=0.391), and T helper cells (R=0.316) (p<0.001), and a negative correlation with NK CD56^bright^ cells (R=-0.540), pDCs (R=-0.487), and NK cells (R=-0.43) (**Figures 8F, G**).

## 4 Discussion

Since irregular vaginal bleeding is an early symptom of EC, most patients are diagnosed at an early stage. Although early diagnosis confers some survival advantage, the annual mortality rate due to EC is still high (4, 26), which warrants the identification of novel prognostic biomarkers. The aim of this study was to identify the prognostic PCD-associated genes in EC in order to construct a predictive model to guide clinical decision-making. A total of 43 PCD-related genes were associated with the prognosis of EC patients, and two distinct clusters were identified.

The patients in Cluster B generally had higher tumor purity, higher T stage, and worse prognosis, while Cluster A was associated with higher ESTIMATE scores, ImmuneScore, and StromalScore. The ImmuneScore is a measure of the percentage of cytotoxic and memory T cells at the tumor core and tumor margin (27), and the StromalScore is indicative of the ratio of stromal cells in the tumor microenvironment (TME) (28). A higher ImmuneScore or StromalScore suggests the presence of more immune substances or matrix components in the TME. Thus, Cluster B was associated with a general decrease in stromal and immune cells and an overall increase in tumor purity compared to Cluster A, indicating that immune infiltration and tumor purity are determinants of EC development and that enhancing the degree of immune infiltration may slow tumor growth. Furthermore, patients in Cluster A generally expressed genes characteristic of immune cell subsets, while patients in Cluster B showed high expression levels of STAT1 and interferon genes, which suggests an immune active state corresponding to favorable prognosis in Cluster A. Consistent with this, Cluster A showed a significant increase in the infiltration of T cells (such as CD8^+^ T cells, cytolytic activity, inflammation-promoting, and T cell co-stimulation) compared to Cluster B. On the other hand, Cluster B showed a significant increase in aDCs, para-inflammation, and type I IFN response compared to Cluster A. Furthermore, WGCNA indicated that the brown module has the strongest correlation with the clusters, and the genes in the brown module are upregulated in Cluster B. The WGCNA algorithm is a powerful tool for identifying co-expressed gene modules and their relationships with phenotypic traits. One of the main advantages of WGCNA is that it can handle large-scale gene expression data sets and identify biologically meaningful gene modules that are associated with specific traits or conditions. Additionally, WGCNA can be used to identify key hub genes that play important roles in regulating gene expression networks. Overall, WGCNA is a valuable tool for understanding the complex relationships between genes and phenotypes. Thus, the brown module was thought to be reliable. In addition, GO analysis showed that the 43 prognostic PCD-related genes were negatively regulated in the pathway of “lipids and atherosclerosis”. Based on these findings, we hypothesize overexpression of PCD-related genes may allow cancer cells to proliferate rapidly by accelerating lipid metabolism.

A LASSO-based PCD-related gene signature comprising 11 genes, including *GZMA, ASNS, GLS, PRKAA2, VLDLR, PRDX6, PSAT1, CDKN2A, SIRT3, TNFRSF1A*, and *LRPPRC*, was developed to predict the OS of EC patients. The LASSO algorithm is a type of linear regression that is used for feature selection and regularization. One of the main advantages of the LASSO algorithm is that it can help prevent overfitting by shrinking the coefficients of less important variables to zero. This can lead to a more parsimonious model that is easier to interpret and less prone to errors. Additionally, the LASSO algorithm can handle high-dimensional data sets with many variables, which can be useful in fields like genetics and finance. The PCD-related gene signature showed good performance in the TCGA-UCEC cohort and two external validation cohorts, TCGA-CESC and TCGA-BRCA. This finding strongly supported the accuracy, sensitivity, and specificity of the PCD-related gene signature in predicting prognosis. GZMA is predominantly expressed in the cytosolic granules of NK cells and cytotoxic T-cells. It cleaves gasdermin-B (GSDMB), which releases the pore-forming moiety of *GSDMB* and triggers pyroptosis (29–31). Asparagine synthetase (ASNS) catalyzes the *de novo* synthesis of asparagine by transferring amino groups from glutamine to aspartic acid. Inhibiting ASNS expression in cancer cells impairs nutrient uptake and promotes apoptosis (32). GLS hydrolyzes glutamine to produce glutamate (33), and its inhibition can induce apoptosis in tumor cells (34). PRKAA2 encodes the catalytic subunit of AMPK, a key enzyme that senses cellular energy status (35). VLDLR is a cell surface receptor with multiple functions, such as binding to very low-density lipoprotein and facilitating its endocytosis, which contributes to energy metabolism (36). PRDX6 is a mercaptan-specific peroxidase that reduces hydrogen peroxide and organic hydroperoxide to water and alcohol respectively (37, 38), and can attenuate apoptosis induced by oxygen-glucose deprivation/reoxygenation (39). PSAT1 is a member of the V-class pyridoxal phosphate ester-dependent transaminase family that fuels tumor cells by generating serine. Inhibition of PSAT1 expression can suppress serine synthesis in tumors, thereby inhibiting their growth (40). CDKN2A prevents MDM2-induced degradation of p53, and promotes p53-dependent apoptosis (41). SIRT3 is an exclusive mitochondrial member of the Sirtuin family of class III histone deacetylases, similar to the yeast Sir2 protein. It can eliminate reactive oxygen species, prevent malignant transformation, and inhibit apoptosis (42). TNFRSF1A belongs to the TNF receptor superfamily of proteins that plays a role in TNFα-mediated cell apoptosis and necrosis (7). LRPPRC is a mitochondrial protein that regulates RNA metabolism and transcription. Loss of LRPPRC affects the electron transport chain in the mitochondria, which increases mitochondrial permeability and generation of reactive oxygen species (43). LRPPRC, CDKN2A, PSAT1, PRDX6, PRKAA2, GLS, and ASNS were highly expressed in the high-risk group, while TNFRSF1A, SIRT3, VLDLR, and GZMA showed high expression in the low-risk group. Consistent with our findings, Tian et al. reported high expression of LRPPRC in EC tissues (44). Upregulation of CDKN2A in the extracellular matrix can promote EC progression by releasing cytokines and proteases in the TME (45, 46). Roh et al. demonstrated that silencing EZH2 in EC cells inhibited PRDX6, leading to the activation of the exogenous homocysteine pathway and eventually cell death (47). Zhou et al. found that estrogen activates Grn metabolism in estrogen-sensitive EC, depending on the up-regulation of GLS (48). However, the functions of LRPPRC, PSAT1, PRKAA2, ASNS, TNFRSF1A, SIRT3, VLDLR, and GZMA in EC progression, and the underlying mechanisms, remain to be elucidated.

The PCD risk score was also calculated across 32 types of cancer and showed marked organ specificity. For instance, uterine carcinosarcoma, brain lower-grade glioma, and ovarian serous cystadenocarcinoma had higher PCD risk scores, while uveal melanoma, mesothelioma, and skin cutaneous melanoma usually had lower PCD risk scores. The PCD risk scores of other cancers were similarly distributed. The PCD-related genes are likely overexpressed in the cancers with higher risk scores, and relatively lowly expressed in cancers with lower scores. We found that cancers with higher mutation frequencies in PCD-related genes tended to have lower PCD risk scores. The mutations may affect the normal expression of PCD-related genes, thereby affecting the level of the risk score. Furthermore, mutations in PCD-related genes may reduce their expression in some cancers, leading to dysregulation of cell death pathways and malignant development. CDKN2A was the most frequently mutated PCD gene across all cancer types, and exhibited the most diverse mutation profile, indicating that SNVs in CDKN2A are ubiquitous in multiple cancers.

The EC patients in the high-risk group were older and have more advanced-stage tumors, which corresponded to a worse prognosis. The PCD gene signature exhibited good predictive performance for 1-, 3- and 5-year survival. Furthermore, the prognosis of high-risk patients worsened with age and tumor grade. GSEA results showed that signaling pathways related to DNA replication were abundant in the high-risk group, while the low-risk group was enriched in pathways related to chemotaxis. This indicated that the tumor cells in the high-risk population proliferate actively, while an active immune response characterizes the tumors in the low-risk population. There were remarkable discrepancies in the distribution of specimens between high-risk and low-risk groups for the four different types of immunization. The patients in the high-risk group were sensitive to AT3148, elephantirin, I-BET-762, and Niraparib, suggesting their potential clinical applicability in EC patients. We also established a nomogram consisting of age, stage, grade, and risk score, which predicted the 1-, 3- and 5-year survival rates with high accuracy.

The PCD genes also showed a significant correlation with several proliferation-related genes. WNT5A is known to regulate the proliferation, invasion, and metastasis of tumor cells. In a previous study, we found that low expression of WNT5A in gastric cancer tissues was significantly associated with the invasion and metastasis of tumor cells, and poor prognosis (49, 50). MKI67 is a typical marker of cell proliferation that remains on the single mitotic chromosome after the breakdown of the nuclear membrane. It is expressed at low levels in EC tissues (51). Mutations in the CTNNB1 gene promote the development of esophageal cancer by upregulating the Wnt/beta-catenin pathway and the downstream target genes (52). CDH1 encodes E-cadherin, an epithelial marker that regulates cell adhesion, migration, and proliferation. It is downregulated during epithelial-mesenchymal transformation (EMT), which is the driver of tumor cell metastasis (53). Low expression of E-cadherin is linked to worse prognosis and survival (54). No study so far has reported any interaction between LRPPRC and these proliferation-related genes in EC. Based on the expression patterns and clinical association of LRPPRC, this gene is likely a risk factor in EC patients and therefore a potential therapeutic target. LRPPRC showed a strong positive correlation with GLS, PRKAA2, and PSAT1. There is a possibility of a synergistic interaction between these genes in EC development, which warrants further research. Wang et al. have identified biomarkers in different tumors by combining computational biology methods such as WGCNA, opening up more possibilities for researching tumorigenesis mechanisms (55, 56). LRPPRC expression showed a positive correlation with Th2 cells and a negative correlation with NK CD56^bright^ cells, which may accelerate tumor progression. High expression of LRPPRC may inhibit NK cell activity, thereby suppressing the immune response and promoting cancer progression. Further studies are needed to assess the role of LRPPRC in tumorigenesis and development, and the underlying molecular mechanisms involved in programmed cell death. Single-cell sequencing (scRNA-seq) has been widely used to explore the mechanisms and biomarkers of gynecological tumors (57, 58). In our subsequent study, we will focus on the mechanism of LRPPRC in EC development through the scRNA-seq approach.

## 5 Conclusion

PCD-related genes are involved in the development of EC and can predict patient prognosis. We developed a PCD-related cluster system for discriminating EC patients with different prognoses. We also constructed an 11-gene PCD-related signature with high predictive performance in prognosis, mutation, and drug response. LRPPRC, an adverse prognostic gene in EC and a member of the model genes, could predict the clinical status and immune infiltration level of EC patients. Our findings provide new insights into the mechanisms underlying EC development and highlight potential therapeutic targets.

## Data availability statement

The original contributions presented in the study are included in the article/**Supplementary Material**. Further inquiries can be directed to the corresponding authors.

## Ethics statement

The studies involving human participants were reviewed and approved by Department of Obstetrics and Gynaecology, Guangzhou Women and Children’s Medical Center, Guangzhou Medical University. The patients/participants provided their written informed consent to participate in this study.

## Author contributions

KG contribute to the method and the results, JX wrote the manuscript. Other authors confirmed their contribution to this research. All authors contributed to the article and approved the submitted version.

## Supplementary material

Supplementary Figure 1 External validation of PCD-related genes signature. **(A)** K-M curves for OS in high-risk and low-risk patient groups. **(B)** ROC analysis to evaluate the performance of signature in 1-year, 3-year, and 5-year prognoses. **(C)** Calibration curves to evaluate the performance of signature in 1-year, 3-year, and 5-year prognoses. **(D)** K-M curves for OS in high-risk and low-risk patient groups. **(E)** ROC analysis to evaluate the performance of signature in 1-year, 3-year, and 5-year prognoses. **(F)** Calibration curves to evaluate the performance of signature in 1-year, 3-year, and 5-year prognoses.
