# Supplementary material for: A novel machine learning-based programmed cell death-related clinical diagnostic and prognostic model associated with immune infiltration in endometrial cancer
Source: Front Oncol. 2023 Jul 18;13:1224071. doi: 10.3389/fonc.2023.1224071 (PMC10393255; doi:10.3389/fonc.2023.1224071)
Supplement: Supplementary Figure 1 — External validation of PCD-related genes signature. (A) K-M curves for OS in high-risk and low-risk patient groups. (B) ROC analysis to evaluate the performance of signature in 1-year, 3-year, and 5-year prognoses. (C) Calibration curves to evaluate the performance of signature in 1-year, 3-year, and 5-year prognoses. (D) K-M curves for OS in high-risk and low-risk patient groups. (E) ROC analysis to evaluate the performance of signature in 1-year, 3-year, and 5-year prognoses. (F) Calibration curves to evaluate the performance of signature in 1-year, 3-year, and 5-year prognoses. [file Image_1.pdf]

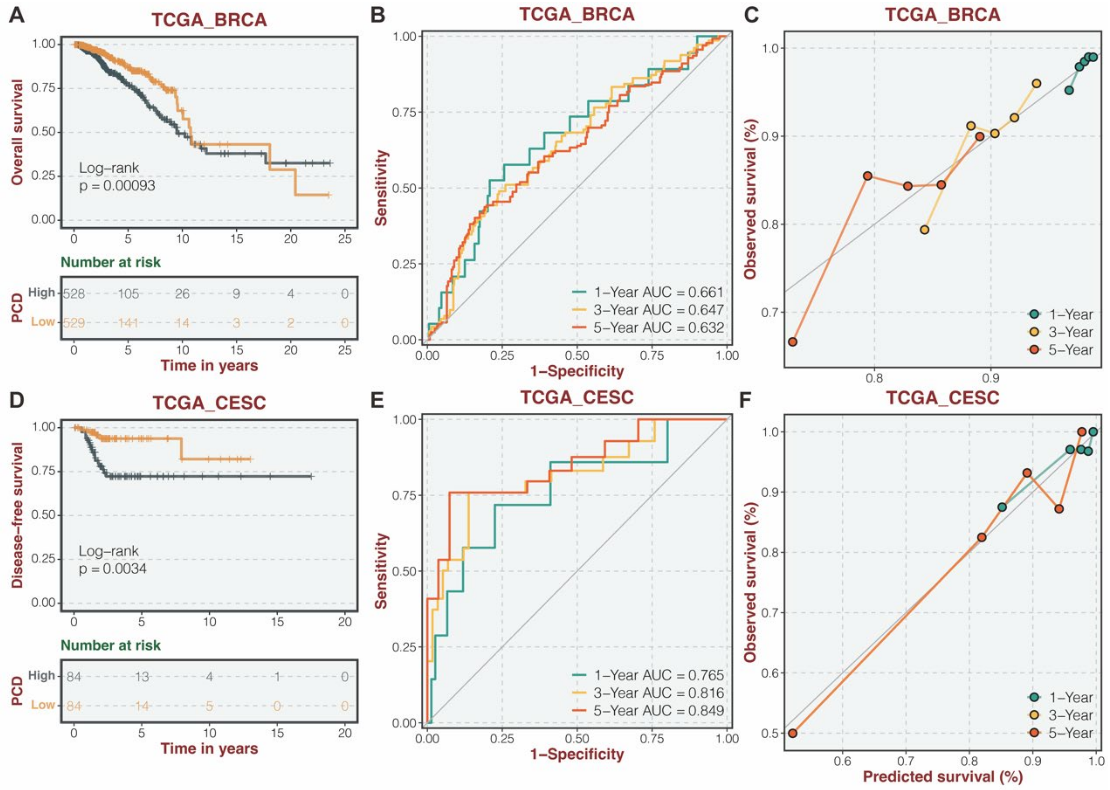

Figure S1: External validation of PCD-related genes signature. (**A**) K-M curves for OS in high-risk and low-risk patient groups. **(B)** ROC analysis to evaluate the performance of signature in 1-year, 3-year, and 5-year prognoses. (**C**) Calibration curves to evaluate the performance of signature in 1-year, 3-year, and 5-year prognoses. (**D**) K-M curves for OS in high-risk and low-risk patient groups. **(E)** ROC analysis to evaluate the performance of signature in 1-year, 3-year, and 5-year prognoses. (**F**) Calibration curves to evaluate the performance of signature in 1-year, 3-year, and 5-year prognoses.
